# Positron Emission Tomography-Computed Tomography Imaging of Selective Lobar Delivery of Stem Cells in *Ex Vivo* Lung Model of Mechanical Ventilation

**DOI:** 10.1089/jamp.2022.0013

**Published:** 2023-02-09

**Authors:** Luiz Maracaja, Ashish K. Khanna, Sean V. Murphy, Danielle L.V. Maracaja, Magan R. Lane, Oula Khoury, Josh Tan, Naresh Damuka, Freda F. Crawford, Joseph A. Bottoms, Mack D. Miller, David W. Kaczka, James Eric Jordam, Kiran Kumar Solingapuram Sai

**Affiliations:** ^1^ Department of Anesthesiology, Wake Forest Baptist Medical Center–Wake Forest School of Medicine, Winston-Salem, North Carolina, USA.; ^2^ Outcomes Research Consortium, Cleveland, Ohio, USA.; ^3^ Wake Forest Institute for Regenerative Medicine, Wake Forest School of Medicine, Winston-Salem, North Carolina, USA.; ^4^ Department of Pathology, Wake Forest Baptist Medical Center–Wake Forest School of Medicine, Winston-Salem, North Carolina, USA.; ^5^ Department of Cardiothoracic Surgery, Wake Forest Baptist Medical Center–Wake Forest School of Medicine, Winston-Salem, North Carolina, USA.; ^6^ Department of Radiology, Wake Forest Baptist Medical Center–Wake Forest School of Medicine, Winston-Salem, North Carolina, USA.; ^7^ Department of Anesthesia, The University of Iowa Hospital and Clinics, The University of Iowa, Iowa City, Iowa, USA.

**Keywords:** lung cell therapy, mechanical ventilation, selective lobe delivery, lung health, pulmonary delivery platform

## Abstract

**Introduction:**

The delivery of cell therapies may be an important frontier to treat different respiratory diseases in the near future. However, the cell size, delivery conditions, cell viability, and effect in the pulmonary function are critical factors. We performed a proof-of-concept experiment using *ex vivo* lungs and novel subglottic airway device that allows for selective lobar isolation and administration of drugs and biologics in liquid solution deep into the lung tissues, while simultaneously ventilating the rest of the lung lobes.

**Methods:**

We used radiolabeled cells and positron emission tomography-computed tomography (PET-CT) imaging to demonstrate the feasibility of high-yield cell delivery to a specifically targeted lobe. This study proposes an alternative delivery method of live cells labeled with radioactive isotope into the lung parenchyma and tracks the cell delivery using PET-CT imaging. The technique combines selective lobar isolation and lobar infusion to carry large particles distal to the trachea, subtending bronchial segments and reaching alveoli in targeted regions.

**Results:**

The solution with cells and carrier achieved a complete and homogeneous lobar distribution. An increase in tissue density was shown on the computed tomography (CT) scan, and the PET-CT imaging demonstrated retention of the activity at central, peripheral lung parenchyma, and pleural surface. The increase in CT density and metabolic activity of the isotope was restricted to the desired lobe only without leak to other lobes.

**Conclusion:**

The selective lobe delivery is targeted and imaging-guided by bronchoscopy and CT to a specific diseased lobe during mechanical ventilation. The feasibility of high-yield cell delivery demonstrated in this study will lead to the development of potential novel therapies that contribute to lung health.

## Introduction

The alveolar membrane of the mammalian lung is composed of a thin and delicate cellular layer, presenting a large surface area for the exchange of gases between the atmosphere and the blood. The tracheobronchial tree, with its multiple bifurcating airway segments, transports these gases to and from the alveoli. This branching network also constitutes a defense mechanism against aspiration of particulate matter, environmentally inhaled particles, and aerosols that may carry microorganisms or environmental pollutants.^1^

The inhalation of small particles has been used for many decades as the primary route for delivery of aerosolized therapeutic agents for treatment of various diseases and syndromes of the respiratory system, including asthma, chronic obstructive pulmonary disease, and pulmonary hypertension.^2^ Inhalational delivery may offer distinct advantages over enteral or intravenous (IV) delivery routes, given its more rapid onset with reduced potential for systemic side effects.^3,4^ However, particle deposition in the alveoli and lower respiratory tract depends on several factors, including particle size, inspiratory flow velocity, inspiratory pause duration, regional ventilation heterogeneity, and whether ventilation is spontaneous or assisted with positive pressure.^5^ Given such factors, aerosolized therapeutic agents may be deposited within the upper airway and tracheobronchial tree, where they can be systemically absorbed.^6^

Aerosol delivery is most efficient during spontaneous breathing, especially with nebulized particle sizes ranging from 2 to 10 μm, high inspiratory flows (120 mL sec^−1^), long inspiratory pauses, and relatively homogeneous regional ventilation.^7^

The highly asymmetric branching pattern of the tracheobronchial tree is an important determinant of early particle impaction on airway walls.^7^ In the human lung, there are ∼24 generations of bifurcations until the respiratory bronchi and alveolar microenvironment are reached.^8^ As inhaled particles travel down the tree, successive bifurcations result in abrupt changes in airway segment cross-sectional area and flow direction, thus causing turbulent flow patterns that direct particles against the airway walls.^9^ Accordingly, the same defense mechanism that prevents the inhalation of toxins or bacteria from reaching the alveoli also hinders the delivery of inhaled therapeutics.

Thus, current options for inhaled delivery of aerosols during mechanical ventilation are limited in efficacy. While liquid delivery of inhaled therapeutics may allow for improved penetration into the periphery, total liquid emersion of the lung requires highly specialized equipment,^10^ very expensive perfluorocarbon solution, and it may be too risky for a patient with poor or borderline pulmonary function. Nonetheless, the delivery of cell therapies may be an important option for the treatment of various respiratory diseases. However, the cell size, delivery conditions, cell viability, and airway tree morphometry remain important factors that often make the aerosol route not a viable option to reach the lung parenchyma at the alveolar level.

In this study, we propose an alternative method for inhalational delivery in the lung, which combines lobar isolation and liquid infusion to carry large particles across the trachea and subtending bronchial segments, to reach alveoli in targeted regions. Specifically, we investigated the delivery of live cells in a large scale to the lung parenchyma, which is not possible using traditional aerosolization methodologies. We used radiolabeled living cells to determine whether our approach is capable of delivering large particles to the parenchyma and to understand the advantages and limitations of selective lobar delivery.

## Methods

**FIG. 1.**
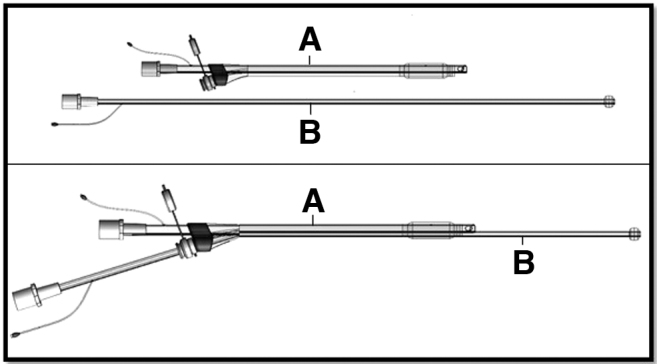
Apparatus for selective lobar delivery during mechanical ventilation. On top, the tracheal tube **(A)** and lobar tube **(B)** are shown separately. On the bottom, the lobar tube **(B)** is placed through the sheath along the tracheal tube **(A)**.

Three *ex vivo* pilot experiments were performed using a novel subglottic airway device that allows for selective lobar isolation and administration of drugs and biologics in liquid solution deep into the lung tissues, while simultaneously ventilating the rest of the lung (Fig. 1). We demonstrated the feasibility of high-yield cell delivery to specifically targeted lobes using radiolabeled cells and positron emission computed tomography (PET-CT) imaging.

### Stem cell preparation

Stem cells derived from placental tissue were isolated, characterized, and cryopreserved in the Regenerative Medicine Clinical Center located within the Wake Forest Institute for Regenerative Medicine. This cell line has been developed under current Good Manufacturing Practices in accordance with regulations for clinical applications and was banked and characterized under the Food and Drug Administration guidelines for rapid translation of research into clinical trials, as previously described.^11^ Briefly, placental stem cells (PLSCs) were grown at 37°C in a 5% CO_2_ humidified atmosphere. Before use, cryopreserved cells were thawed, cultured for 4 days in alpha-minimal essential medium (alpha-MEM) supplied with AmnioMAX media, and were supplied with 18% fetal bovine serum and 1% penicillin/streptomycin. PLSCs were detached using 0.05% or 0.25% [for mesenchymal stem cell basal medium (BM-MSCs)] trypsin, and viability was assessed using trypan blue.

### A-549 cells

The A-549 cell line consists of cultured alveolar basal epithelial cells isolated from human adenocarcinoma. These cells contain lamellar bodies, an intracellular organelle for the transport and storage of surfactants, similar to type II pneumocytes. Cultured cells were plated 5 days before the experiment into six-well plates or six T-225 flasks culture media, yielding ∼6 million cells or 60 million cells, respectively, on the day of the experiment. Daily inspection with microscopy was performed to confirm the cells were viable and uncontaminated. The cell diameter of PLSCs and A-549 cells ranges from 17 to 30 μm and from 10 to 14 μm, respectively.

### Radiochemistry: 18F-FDG uptake

*In vitro* uptake of the radiotracer fluorodeoxyglucose (18F-FDG; PETNET Solution, Inc., NC) was performed following our previous protocol with slight modification.^12^ Briefly, cells were cultured in tissue culture plates. Before adding the radiotracer, the cells were carefully washed with phosphate-buffered saline (PBS) in a sterile fume hood and treated with 18F-FDG. After incubation for 2 hours at 25°C, the cells were washed in PBS (three times), and the residual washes were separately collected for quantifying the efficacy of radiotracer binding. All the cells were then treated with trypsin-ethylene diamine tetraacetic acid (trypsin-EDTA) (0.05%) solution to manage the final cell pellet. The radioactivity from the washing fractions and desired cell pellet fraction were measured using a standard Geiger counter (Table 1). 18F-FDG binding yield was ∼5.3% (decay corrected to end of labeling). The complete procedure, from cell washing to final radioactive aliquot administration, took about 140 minutes. Finally, 18F-FDG-labeled cells suspended in PBS were used in the PET-CT acquisition protocol.

**Table 1.** Approximate Number of Cells Used for 18F-FDG Radiolabeling, Administered Dose, and the Resultant Radioactive Uptake Calculated as Standard Uptake Values in the Associated Lung Lobes

Experiment	1-A549	2-Stem cell	3-A549
No. of cells	6 million	60 million	60 million
Dose MBq	3.7	8.806	0.95
SUVs (lung lobe)	58.0087	56.0493	36.9464

18F-FDG, fluorodeoxyglucose; SUVs, standard uptake values.

### Lung preparation and imaging

To demonstrate successful delivery of the cells to the targeted lobe, we used combined PET-CT imaging on a single gantry in the same session. The superposed images allow for functional imaging of the spatial distribution, metabolic, and/or biochemical activity in a specified region of interest.^13^ Experiments were performed in three 65 kg Yorkshire Landrace pigs. Animal tissues were procured following euthanasia from protocols approved by our institutional animal care and use committee. The trachea, heart, and lungs were resected *en bloc* immediately after euthanasia under general anesthesia and placed in ice. The trachea was then intubated, and the lungs were inflated to 20 cmH_2_O of continuous pressure. Lobar intubation was performed with fiber-optic bronchoscopy and confirmed manually by external bronchial palpation.

The left lower lobe was chosen for targeted delivery of cells for the first two experiments, due to its resemblance to human anatomy and less anatomical variation on the left side. The lobar tube was positioned on the right lower lobe on for the third experiment. Pigs have upper, middle, and lower lobes similar to humans, but pigs have a unique small accessory lobe that is posterior and inferiorly located inside the chest cavity—the pig's right upper lobe takes off from the trachea. Despite having a different tracheal and bronchial origin of the lobe bronchi, the right lower lobe volume and location inside the chest cavity are similar to human anatomy.

The lobar tube was connected to a ventilator, and the volume of the lobe was measured at the end of inspiration with no visual atelectasis and inflated to 20 cmH_2_O. The bronchial and tracheal tubes were then clamped to maintain expansion. The excised lungs were imaged using a GE Discovery PET-CT scanner at the Translational Imaging Program at Wake Forest PET Research Center. This system is designed for molecular imaging of different tracers superimposed on high-resolution computed tomography (CT) images. Two PET imaging bed positions with 3-minute acquisition times and CT attenuation correction were used to obtain the images. Two- and three-dimensional image reconstruction may be rendered as a function of TeraRecon software (TeraRecon, Durham, NC) and control system. Table 1 summarizes the number of cells used in the 18F-FDG radiolabeling, the final dose injected to the lobes, and the associated radioactive uptake quantified with standard uptake values (SUVs).

### Cell delivery

The lungs were positioned and stabilized on the scanner table. The tracheal lumen was connected to an anesthesia machine circuit with a constant pressure of 15 cmH_2_O. The lobar lumen was open to atmosphere and then aspirated with a long suction catheter to remove secretions and to provide full atelectasis of the lower lobe. We premeasured the volume of saline to fill the lobar tube, and we diluted the cell concentration immediately before administration. A special delivery system was mounted on the PET-CT table to allow the delivery of pressurized fluid to the lower lobe without operator exposure to the nuclear isotope.

The solution with the cells was slowly injected to minimize bubble formation, and completely filled the lobe and lobar tube up to the connector level. The lobar tube was then immediately connected to the delivery system that allowed for a volume excursion profile similar to that of a mechanical breath, although with a liquid volume of ∼350 mL. After five such fluid volume cycles, the lobar tube was clamped, and PET-CT imaging was obtained. The clamp on the fluid-filled tube was necessary to keep the lobe in position to obtain stable PET-CT imaging.

## Results

**FIG. 2.**
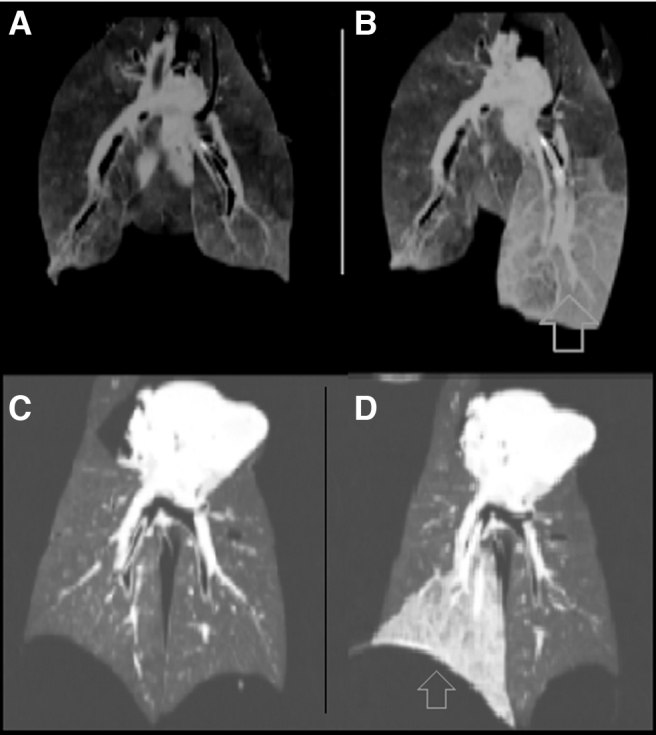
Coronal CT imaging of the lungs before **(A, C)** and after **(B, D)** lobar A-549 cell delivery. After the delivery, cells and flush showing liquid density lobe bronchi, and higher density of the parenchyma with preserved tissue architecture (blue arrow), **(B)** left lower lobe, **(D)**right lower lobe. CT, computed tomography.

**FIG. 3.**
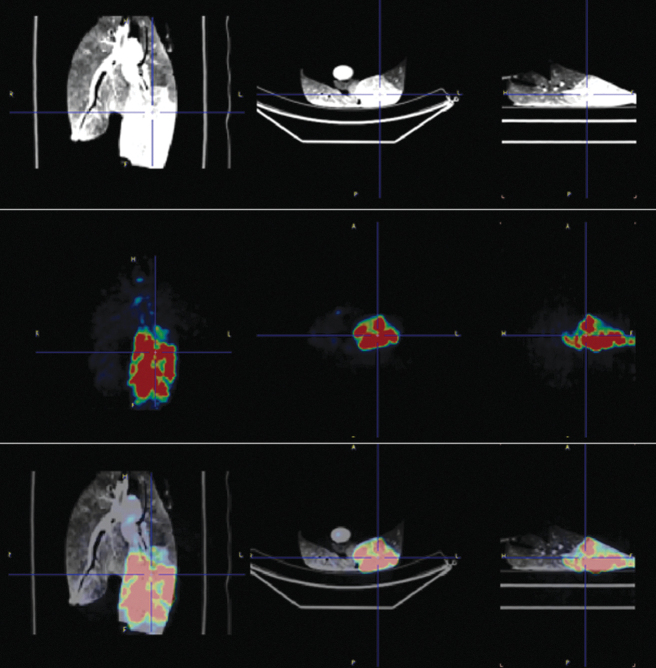
Imaging of left lower lobe delivery of 18F-FDG-labeled cells experiment. From the left to right: coronal, axial, and sagittal planes. Top row: CT imaging. Middle row: PET imaging. Bottom row: Fused CT and PET imaging showing the isotope-labeled cell distribution. 18F-FDG, fluorodeoxyglucose; PET, positron emission tomography.

**FIG. 4.**
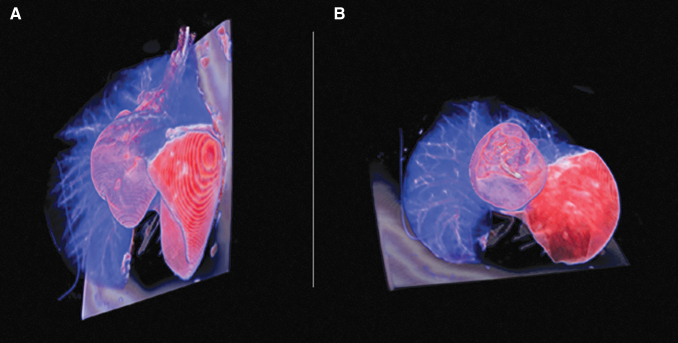
Three-dimensional rendering of 18F-FDG-labeled placental stem cells delivered in the left lower lobe. **(A**) Perspective view. **(B**) Bottom view. Fused PET-CT image reconstruction showing in blue the normal lung parenchyma, and in *red* the isotope-labeled cells distributed across the entire left lower lobe.

Cell viability was confirmed with microscopy before the F18-FDG uptake, and the radiation count was measured before the dilution on the lobar tube volume. The radiation count was proportional to the cell count, yielding fairly low absolute radiation level (Table 1). The SUV numbers obtained were proportional to the radioactive dose administered. High SUVs associated with the cells in the lung lobes clearly demonstrated both selectivity and specificity of our imaging technique. The solution with cells and carrier achieved a fairly homogeneous increase in tissue density on the entire lobe shown on the CT scan (Fig. 2). The PET-CT imaging demonstrated retention of the activity at central and peripheral lung parenchyma (Figs. 3 and 4), not restricted to the tracheal bronchial tree. The increase in CT density and metabolic activity of the isotope was restricted to the desired lobe only, without evidence of leakage to other lobes.

## Discussion

Current delivery methods for inhalation therapies to the lung periphery face substantial pharmacodynamic challenges. The complex anatomy of the tracheobronchial tree, as well as differences in regional perfusion and absorption capacity of the lung, limit therapeutic options that can be administered by inhalation.^14^ At present, respiratory failure is treated with mechanical ventilatory support, IV drugs, and nebulization of pharmacological agents with the potential to reduce disease progression and restore pulmonary function.

Delivery of aerosolized particles may also be substantially reduced during invasive mechanical ventilation. A previous study using 18m Krypton scintigraphy and aerosolized technetium showed that only 18% of a nebulized dose delivered during mechanical ventilation in an intubated porcine lung actually reaches the tracheobronchial tree, with most of these particles being deposited on the central airway walls. The remaining 82% were distributed on the endotracheal tube, ventilator circuit, and filters. A negligible amount, if any, reached the lung periphery.^14^ Such observations speak to the current limitations of aerosolized delivery of therapeutics during invasive mechanical ventilation.

Aerosolized delivery has particular limitations in treating heterogeneous lung diseases, such as acute respiratory distress syndrome (ARDS), which predominantly affects gravity-dependent lung regions. ARDS is associated with surfactant dysfunction, alveolar collapse, and airspace edema, which arise from inflammatory sources. The syndrome creates a significant heterogeneity of mechanical properties of the parenchyma that maldistributes regional ventilation.^15^ Lung zones with minimal or no regional ventilation will not effectively receive aerosolized delivery, whereas unaffected zones with better compliance may receive most of the flow during aerosolized delivery at the airway opening.

Our group has demonstrated the feasibility of selective lobar ventilation in a previous report.^16^ By contrast, liquid solutions can be very effective in reaching less compliant alveoli if properly administered. We know from research models of ARDS and liquid ventilation that alveolar surfactant can be extracted from the alveoli with lung lavage with saline solutions.^17^ However, the deposition of large liquid volumes can further deplete surfactant, resulting in more alveolar collapse and hypoxemia.

Pulmonary surfactant delivery is crucial to the management of neonates with hyaline membrane disease.^18^ Unfortunately, studies using surfactant in adult patients with ARDS have not shown significant benefit,^19^ likely due to the aforementioned limitations of the delivery method. Our selective lobar delivery technique overcomes many of the limitations of aerosol delivery, such as particle size and heterogeneous ventilation distribution. Accordingly, lobar delivery of high fluid volumes could potentially resurface the alveolar membrane in collapsed lung regions.

The effective delivery of large particles or cells into the lungs is not achievable with current delivery systems. Delivery during inhalation is difficult because of the large cell size and their potential for desiccation. Large particles or cells contained within small fluid volumes are likely to be heterogeneously distributed in the diseased lung and thus may not reach targeted alveoli. Indiscriminate infusion of large fluid volumes into the lungs can potentially impair ventilation and oxygenation due to surfactant dysfunction and flooding of nontargeted lobes simultaneously, with little therapeutic distribution of cells to targeted areas with increased surface tension and reduced compliance. Targeted delivery to a single lobe through catheter of small diameter or the channel of a bronchoscope would likely not generate enough flow and pressure to bring the cells across the tracheobronchial bifurcations to reach the alveoli.

This study was performed as proof of concept to demonstrate that cells and larger particles can reach the lung parenchyma using a high-flow, positive-pressure, and high-volume lobar liquid-based system. Hopefully, this technique could be used to provide topical treatment in high concentration, with less systemic toxicity for various disease processes, such as lobar pneumonia, cavitary lesions,^20,21^ lung cancer,^22^ sarcoidosis,^23^ and interstitial lung disease.^24^ Ultimately, this method is capable of efficiently delivering gene^25,26^ and cell therapies for pathologies that cause tissue necrosis with the destruction of lung parenchyma. Cell and gene therapies have the potential for breakthrough discovery for new therapies in various disease processes, particularly in regenerative medicine and oncology.

Adoptive cell transfer, cellular adoptive immunotherapy, and T-cell transfer therapy are part of the treatment of certain hematologic malignancies and infections. Because the lungs have a vital function that cannot be interrupted, the use of therapeutics has been limited by a delivery route that will minimally impact gas exchange. Selective lobe delivery will likely not interfere significantly with the gas exchange, particularly under the controlled conditions of general anesthesia and supplemental oxygen. Regenerative medicine uses many well-developed cell therapy approaches to treat human diseases. Stem cells from different tissue sources such as bone marrow, umbilical cord, amniotic fluid, and placenta, among others, have been investigated for their potential therapeutic application for transplantation in blood and autoimmune disorders, skin grafts for severe burn, and corneal injuries.^27^

PLSCs carry very strong anti-inflammatory properties and have the potential to engraft the injured tissue site if adequately delivered. The delivery methods for cell therapy include IV injection in the peripheral blood,^28^ direct deposition at site of injury, or direct placement or delivery into new scaffolds,^29^ among others. For pulmonary applications, aerosol delivery limits the delivery of PLSCs due to cell size, mass, and need for a liquid vehicle to maintain cell viability and suspension to prevent desiccation. Selective lobar delivery in cell media could better maintain viability and delivery with much higher cell count on the alveolar surface. Direct lobar delivery could also offer advantages for other therapies, such as brachytherapy microspheres for cancer treatment.

Lung cancer is associated with the highest mortality rate among solid cancers.^30^ Recent studies have shown that cell therapies^31^ offer potential benefits as an adjuvant to surgical and other treatment modalities. The lobe or segment delivery would be ideal to deliver radiation therapy and prevent undesirable radiation pneumonitis to surrounding normal lung tissue. The placement and stabilization of the lobar tube depend on the landing zone for the cuff. The mainstem bronchi and lower lobe bronchi have a good landing zone, unlike the upper lobes, which have short bronchi before bifurcation into segments. The delivery to the upper lobes can be performed through the tracheal lumen while ventilating the distal bronchial tubes, placed in the mainstem (contralateral lung) and lower lobe (ipsilateral).

In this study, we were limited by *ex vivo* constraints, such as the inability to study the spatial and temporal distribution of radiolabeled cells after initial delivery. The FDG cell uptake yield was lower than expected but sufficient to track the cell distribution with PET-CT. Additional limitations for clinical use of selective lobar delivery are the requirement of general anesthesia, intubation, bronchoscopy, specialized equipment, and exposure to ionizing radiation if CT scan is necessary.

Moreover, the safety and efficacy of our lobar delivery technique require further testing. The ability to isolate a lung lobe and create atelectasis allows assessment of the contribution of that lobe to mixed arterial oxygenation before the delivery. Lobar atelectasis before the infusion and positive pressure delivery simulating a respiratory cycle will likely be beneficial for tissue cell distribution during the delivery. We believe that the *ex vivo* testing was necessary because it allowed direct visualization of the different lung lobes during the delivery, but also to test the feasibility of this method and rehearse the technique before test in live animals. Cell delivery in live subjects and a radiotracer with a longer half-life will be necessary to confirm the reproducibility of our results and confirm cell uptake within the pulmonary lobe.

This novel method is not restricted by the limitations that are typical of current aerosol delivery systems. The selective lobe delivery allows for the use of a higher concentration and better distribution of the desired therapy into the lung parenchyma. The feasibility of radiolabeled cell delivery demonstrated in this study will lead to the development of potential novel therapies that contribute to lung health.

## Notes

Reviewed by:

Myrna Dolovich

Gerald Smaldone
